# Diffusion of a collaborative care model in primary care: a longitudinal qualitative study

**DOI:** 10.1186/1471-2296-14-3

**Published:** 2013-01-04

**Authors:** Isabelle Vedel, Veronique Ghadi, Matthieu De Stampa, Christelle Routelous, Howard Bergman, Joel Ankri, Liette Lapointe

**Affiliations:** 1 Solidage, McGill University - Université de Montréal Research Group on Frailty and Aging - Lady Davis Institute, Jewish General Hospital, H466, 3755, Ch. Côte Ste Catherine, Montreal, Québec H3T 1E2, Canada; 2 Santé Vieillissement research group, Versailles St Quentin University, 49 rue Mirabeau, Paris, 75016, France; 3 Management Institute, Ecole des Hautes Etudes en Santé Publique, Avenue du Professeur Léon-Bernard - CS 74312, Rennes cedex, 35043, France; 4 Department of Family Medicine, McGill University, 515-517 av. des Pins Ouest, Montreal, Quebec, H2W 1S4, Canada; 5 Desautels Faculty of Management, McGill University, 1001 Sherbrooke St. West, Montreal, Quebec, H3A 1G5, Canada

**Keywords:** Primary care, Primary care physician, Nurses, Chronic disease, Collaboration, Health service research, Diffusion of innovation

## Abstract

**Background:**

Although collaborative team models (CTM) improve care processes and health outcomes, their diffusion poses challenges related to difficulties in securing their adoption by primary care clinicians (PCPs). The objectives of this study are to understand: (1) how the perceived characteristics of a CTM influenced clinicians' decision to adopt -or not- the model; and (2) the model's diffusion process.

**Methods:**

We conducted a longitudinal case study based on the Diffusion of Innovations Theory. First, diffusion curves were developed for all 175 PCPs and 59 nurses practicing in one borough of Paris. Second, semi-structured interviews were conducted with a representative sample of 40 PCPs and 15 nurses to better understand the implementation dynamics.

**Results:**

Diffusion curves showed that 3.5 years after the start of the implementation, 100% of nurses and over 80% of PCPs had adopted the CTM. The dynamics of the CTM's diffusion were different between the PCPs and the nurses. The slopes of the two curves are also distinctly different. Among the nurses, the *critical mass* of adopters was attained faster, since they adopted the CTM earlier and more quickly than the PCPs. Results of the semi-structured interviews showed that these differences in diffusion dynamics were mostly founded in differences between the PCPs' and the nurses' perceptions of the CTM's *compatibility* with norms, values and practices and its *relative advantage* (impact on patient management and work practices). Opinion leaders played a key role in the diffusion of the CTM among PCPs.

**Conclusion:**

CTM diffusion is a social phenomenon that requires a major commitment by clinicians and a willingness to take risks; the role of opinion leaders is key. Paying attention to the notion of a *critical mass* of adopters is essential to developing implementation strategies that will accelerate the adoption process by clinicians.

## Background

In primary care, many patients have multiple, interacting, and compounding physical, psychological, and social problems [1,2]. In order to meet these multiple needs at an affordable cost for society [3,4], collaborative team models (CTMs) in primary care such as Patient-Centered Medical Home [5,6], Family Health Teams and Family Medicine Groups [7] are increasingly viewed as essential in order to improve the care of patients with chronic diseases.

Although CTMs may improve care processes and health outcomes [5,8-12], implementation of CTMs remains a challenge [7,13-16], particularly in terms of achieving adoption by clinicians and, more specifically, by primary care physicians (PCPs) [12,14,17-19]. If the implementation of CTMs is to be effective and induce the changes required in the care of patients with chronic diseases, a sufficient number of professionals need to adopt them [14,17]. However, the implementation of new forms of professional relationships and collaboration has rarely been addressed, either theoretically or empirically [20]. Thus, despite the worldwide proliferation of CTMs that are being developed and implemented at enormous cost and effort, there is still considerable uncertainty surrounding whether or not and under what conditions health professionals will adopt them. It is therefore important to develop a better understanding of CTM adoption factors and the model’s diffusion process. According to the Diffusion of Innovations Theory [21], diffusion is the process by which an innovation is communicated over time through certain channels to the members of a social system. The success of an implementation is assured if a certain *critical mass* of individuals adopts the innovation (20%-40% of adopters). Beyond this threshold, the diffusion process acquires a momentum of its own. However, all individuals do not adopt an innovation at the same speed depending on their perception of five characteristics of the innovation [21]: *relative advantage, compatibility, simplicity, trialability,* and *observability* (Table 1).

**Table 1:** Perceived characteristics of innovations and their definitions (according to Rogers, 2003)

**Concept**	**Definition**
Observability	The degree to which the results of an innovation are visible* to others
Trialability	The degree to which an innovation may be experimented with on a limited basis
Simplicity	The degree to which an innovation is perceived as not difficult to understand and use
Compatibility	The degree to which an innovation is perceived as being consistent with the existing values, past experiences and needs of potential adopters
Relative advantage	The degree to which an innovation is perceived as better than the idea it supersedes

Note:* In the case of a model of care that is an untangible innovation, we used the following definition: The degree to which the results of an innovation are understandable to individuals or known by the community.

**Figure 1:**
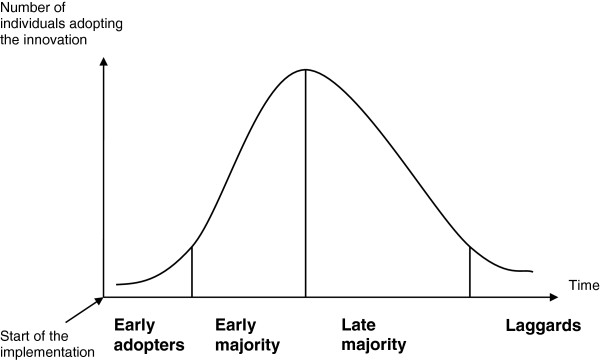
Diffusion of Innovations Theory: normally distributed curve dividing a given population into four adopter categories (adapted from Rogers 2003).

According to the Diffusion of Innovations Theory, *early adopters* adopt the innovation very quickly. Then the number of individuals adopting the innovation grows as it is adopted by *early majority* and *late majority* adopters. Finally, a small number of individuals called *laggards* will resist to the innovation (Figure 1).

Using the Diffusion of Innovations Theory, the objectives of this study were: (1) to analyze how the perceived characteristics of a CTM influenced PCPs’ and nurses’ decision to adopt –or not– the model; and (2) determine the model’s diffusion process.

## Methods

### Study design

A longitudinal case study [22,23] was conducted from September 2006 (the start of the CTM’s implementation) to May 2010 (the end of the study) in a borough of Paris (154,000 inhabitants) where a CTM [24] was being implemented (Table 2).

**Table 2:** **Description of the Collaborative Team Model – COPA**[24]

**Aims of COPA**	This CTM (COPA –Coordination Personnes Âgées), implemented in France (Paris), was designed to provide a better fit between the services provided and the needs of older patients with multiple diseases in order to reduce excess healthcare use, including emergency room (ER) visits and hospitalizations. COPA targets community-dwelling older patients with multiple diseases recruited through their PCP. The originality of the CTM [24] lies in: (1) the integration of Primary Care Physicians (PCPs) and nurses, who act as a core team and collaborate closely in the patient care process (e.g. needs assessments, individualized care plans, follow-up) in order to provide patient-centered and coordinated care; (2) their ad-hoc reliance on the expertise of other community-based professionals (social workers, a psychologist, an occupational therapist, etc.) and (3) the integration of primary medical care and specialized care through the introduction of community-based geriatricians and palliative care specialists (who intervene when a PCP requests advice or a planned hospitalization).
**Context**	In France, PCPs are typically solo practitioners paid on a fee-for-service basis. The nurses are salary workers in community-based services; their role is to provide both case management and direct care. PCPs and nurses in France do not collaborate on a routine basis. They usually do not have access to training programs on inter-professional collaboration.
**Key components of the COPA model**	Under COPA, older patients (65 years old or above) benefit from a multidisciplinary comprehensive geriatric needs assessment, an individual care plan, care management programs, evidence-based protocols, and regular reassessments of their needs. The model integrates health care professionals into a multidisciplinary primary care team. This multidisciplinary primary care team is formed around a two-person team consisting of a nurse-case manager collaborating closely with a PCP in order to provide patient-centered care. For instance, case managers and PCPs develop and implement the care plan and coordinate health and social services across the different settings and among the numerous care providers. Case managers organize inpatient visits and hospital discharge in collaboration with the hospital team. This core team could call on the expertise of other health professionals (various medical specialists, home health nurses, social workers, a psychologist, an occupational therapist, etc.).
**Implementation**	For the implementation of the CTM, all the PCPs and the nurses practicing in this borough of Paris - 175 PCPs and 59 nurses - were identified using a professional directory and contacted. All of them were invited to participate in the model in September 2006. They were free to participate or not.
**Monitoring the implementation process**	A central activity database was maintained by the clinical administrators who recorded data related to: (1) the health professionals’ participation in the model (e.g. date of formal agreement as reported on a consent form) and (2) the collaborative behaviour of the healthcare professionals during the care they provided to each patient (e.g. needs assessment process, individualized care plan development, phone contacts and multidisciplinary meetings).

#### Phase I: The diffusion of the CTM among the 175 PCPs and 59 nurses practicing in the borough

**Figure 2:**
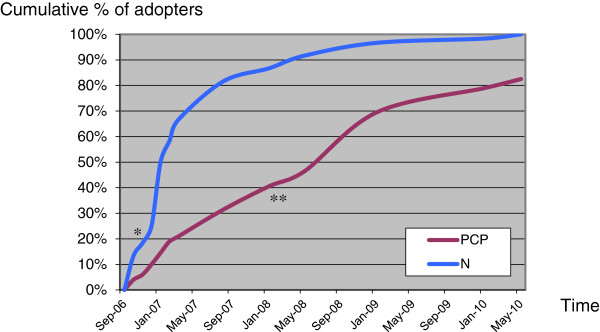
**Curves of diffusion of the Collaborative Team Model among Primary Care Physicians and Nurses. **Legend: PCP: Primary Care Physicians (n=175); N: Nurses (n=59). Note: Inflection point on the curve for nurses (*) and for PCPs (**).

First, we began by plotting diffusion curves representing the diffusion of the CTM among all of the PCPs and nurses practicing in the borough. The dates of each PCP’s and nurse’s formal agreement to adopt the CTM were determined based on information gathered from a central activity database aimed at monitoring the implementation of the CTM (Table 2). These adoption dates were also used to calculate the cumulative number of PCPs and nurses adopting the CTM each month. These data allowed us to plot curves [21] representing the diffusion of the CTM among all of the 175 PCPs and 59 nurses practicing in the borough (Figure 2). Using these curves, it was possible to assign each PCP and nurse to one of the four adopter categories (*early adopters*, *early majority*, *late majority, laggards*).

#### Phase II: Case study

Second, semi-structured interviews were conducted among a representative sample of the four adopter categories to better understand the implementation dynamics. In each of these categories (see Phase I), some PCPs and nurses were selected using a maximum variation sampling strategy (based on gender, age, type of practice) [25] in order to identify the range of views of all four adopter categories. Fifty-seven clinicians were contacted for interviews. Only two PCPs refused to participate; their characteristics were not different from those of the participants. A total of 55 interviews were conducted: 40 with PCPs and 15 with nurses (Table 3). Three researchers trained in qualitative research conducted 45-minute, individual face-to-face interviews at the PCPs’ and nurses’ offices using the same semi-structured interview guide (see Additional file 1). Questions were first developed based on our review of extant literature. They were then validated and refined using three pilot interviews with experts from different domains: public health, family medicine and geriatrics. All the individual interviews were recorded and transcribed verbatim.

**Table 3:** Characteristics of the sample of Primary Care Physicians and Nurses

**Adopter category**	**Occupation**	**Gender**	**Age (mean, years)**
Early adopters	5 Primary Care Physicians (PCPs)	5 males	47.25
	7 Nurses	5 females	44.85
		2 males	
Early majority	11 PCPs	3 females	54.18
		8 males	
	4 Nurses	3 females	40.00
		1 male	
Late majority	15 PCPs	2 females	55.40
		13 males	
	2 Nurses	1 female	51.50
		1 male	
Laggards	9 PCPs	2 females	55.50
		7 males	
	2 Nurses	2 females	59.00

In addition, to support and enhance our understanding of the phenomenon under study, we triangulated our data sources [22,25]. Thus, two researchers spent several days at various community-based health services and attended administrative and multidisciplinary meetings to observe and record key information such as information exchange (e.g. healthcare professionals’ discussions about the CTM) and representative practices (e.g. the use of care plans). The researchers made detailed field notes. Also, documents describing the aims and means of the CTM (minutes, memos) and the healthcare professionals’ participation (activity reports and databases) were coded and analyzed.

We used N’Vivo 8 to code and analyze data using standardized methods of qualitative thematic analysis [25]. First, the analysis began deductively, using the concepts formulated in the Diffusion of Innovations Theory [21]. This was followed by a thematic inductive analysis [25], in which the researchers remained open to new concepts; we used new categories and codes that emerged as important to developing a better understanding of the diffusion process (e.g. characteristics of the opinion leaders and their actions). The data analysis followed an iterative procedure until theoretical saturation [26]. While interviews were our main source of evidence, the data gathered from observation and documentation were also used in order to corroborate, validate and complement the information from the interviews.

### Ethical approval

The study protocol was approved by the Medical Ethics Committee of the Ambroise Paré Hospital (Versailles St. Quentin University).

## Results

### Diffusion curves

The data gathered in Phase I were used to plot curves describing the CTM diffusion among all the 175 PCPs and 59 nurses practicing in the borough. As seen in Figure 2, 3.5 years after the start of the implementation, 100% of nurses and over 80% of PCPs had adopted the CTM. The slopes of the two curves are also distinctly different. Overall, the nurses adopted the CTM earlier (earlier takeoff point on the curve) and faster (higher slope on the curve) than the PCPs. The nurses thus attained a *critical mass* faster (inflexion point of the curve = approximately 20%) compared to PCPs (approximately 40%).

### Perceptions of the model’s characteristics

The analysis of the interviews helped explain the commonalities and differences in the perceptions of the CTM’s characteristics between PCPs and nurses.

#### Common perceptions among PCPs and nurses

Our results indicate that PCPs and nurses shared common perceptions regarding the CTM’s *observability*, *trialability* and *simplicity* (Tables 4 and 5). Overall, the more positive these perceptions were, the earlier the PCPs and nurses adopted the CTM. In terms of *observability*, all the PCPs and nurses had discussed the CTM with other clinicians, which had influenced their opinion. The early adopters and the early majority had discussed it with colleagues who had positive opinions of the CTM. In contrast, the late majority and laggards had discussed it with colleagues who were not convinced of the value of the CTM and who spoke of the implementation of CTMs in negative terms. For *trialability*, the early adopters and the early majority had positive experiences when they tried the CTM with an initial patient. In contrast, the late majority and laggards had poorer first experiences, and decided to postpone adoption. The early adopters and early majority perceived the CTM as *simple*. In contrast, the late majority and laggards generally had difficulties with the CTM, and found it tedious to change their practices due to ingrained habits.

**Table 4:** Primary Care Physicians’ and Nurses’ perceptions of the Collaborative Team Model

		**Early adopters**	**Early majority**	**Late majority**	**Laggards**
**Observability**	**PCPs**	++	+	**-**	**-**
	Nurses	++	+	**-**	**-**
**Trialability**	**PCPs**	**++**	**+**	**-**	**-**
	Nurses	**++**	**+**	**-**	**-**
**Simplicity**	**PCPs**	**++**	**++**	**- -**	**- -**
	Nurses	**++**	**++**	**-**	**- -**
**Compatibility**	***PCPs***	***+***	***+/−***	***- -***	***- - -***
	***Nurses***	***+++***	***++***	***-***	***- -***
**Relative advantage**	***PCPs***	***+***	***+/−***	***-***	***- -***
	***Nurses***	***+++***	***++***	***+/−***	***-***

Legend:

PCPs: Primary care physicians.

+ Positive perception favoring the adoption of the CTM; - Negative perception representing a barrier to adoption.

Note: This table presents a summary of the analysis of the interviews. It highlights (in bold italic) the areas of divergence between PCPs and nurses that may explain the differences of behavior between these two professional groups.

**Table 5:** Selected quotes: Primary Care Physicians’ and Nurses’ perceptions about the Collaborative Team Model

	**Primary Care Physicians (PCP)**	**Nurses (N)**
**Common perceptions among PCPs and Nurses**		
**Observability**	Positive opinion (early adopter, early majority)	Positive opinion (early adopter, early majority)
	*Eg. I talked about [the CTM] a bit with one of my colleagues who has a practice much like mine, and we agreed that it could help us.* PCP, early majority	*Eg. We talked about [the CTM], and everyone thought that it’s a good thing.* N, early adopter
	Negative opinion (late majority, laggards)	Negative opinion (late majority, laggards)
	*Eg. We don’t hear much that is good about the networks that have been implemented. The reports aren’t very good.* PCP, late adopter	*Eg. My colleague will tell you exactly the same thing; (these models) are taking our jobs.* N, laggard
**Trialability**	Positive opinion (early adopter, early majority)	Positive opinion (early adopter, early majority)
	*Eg. I had to invest a bit of time finding [the CTM]. I tested it when I had a chance, and I found it very good.* PCP, early majority	*Eg. I tried [the CTM] once just to see, and it was great. So I hope it will continue.* N, early majority
	Negative opinion (late majority, laggards)	Negative opinion (late majority, laggards)
	*Eg. I proposed a patient, but I guess they didn’t quite meet their criteria… So I didn’t continue; I waited.* PCP, late majority	*Eg. We don’t even work the same way, we don’t have the same approach to work or availability. The first time, they said I’d find all the information there, but when I went, I saw all the faults in the system. So I said no.* N, laggard
**Simplicity**	Positive opinion (early adopter, early majority)	Positive opinion (early adopter, early majority)
	*Eg. It’s going well. When I call, I get a quick callback if I need [the CTM]. Right there I have my solution, right away.* PCP, early adopter	*Eg. [the CTM] really works very simply, (…) it’s highly available, and very responsive. It’s really easy.* N, early adopter
	Negative opinion (late majority, laggards)	Negative opinion (late majority, laggards)
	*Eg. It isn’t easy to change your practices. When we learned how to work on our own, it didn’t come easily.* PCP, late adopter	*Eg. It’s very unclear how it works: a [CTM] can mean anything.* N, late adopter
**Different perceptions among PCPs and Nurses**		
**Compatibility**	Positive opinion but caution (early adopter, early majority)	Strong positive opinion
	*Eg. To the extent that everyone knows their place and helps each other out, that we don’t start seeing encroachment by other specialists or anything like that, then [the CTM] may be a good experience.* PCP, early adopter	*Eg. They’re people with know-how, who don’t take over, who in fact try to respect people in their practices, to express themselves very diplomatically.* N, early majority
	Strong negative opinion (late majority, laggards)	Mild negative opinion (late majority, laggards)
	*Eg. If the case manager centralizes the information, that makes me uncomfortable. Medical information can only move from one physician to another with the patient’s agreement.* PCP, laggard	*Eg. I know that nowadays the style is to produce a lot of paper… We preferred spending our time with the person rather than on doing paperwork.* N, laggard
	*Eg. I imagine that they’re going to impose constraints that don’t fit our practices… We don’t work the same way. So I can’t see how we can develop closer ties.* PCP, laggard	
	*Eg. There are patients that I haven’t seen since. In particular, there’s a woman with dementia. She was being followed by [geriatrician], she didn’t have other needs than to see [geriatrician].* PCP, late majority	
	*Eg. When I learn that a patient I’m following has been asked to take a certain test and I learn it from a member of his family, I’ve got problems with that…* PCP, laggard	
	*Eg. We’ve known our patients for years, and in a few days they’ll change the patient’s treatment without telling us.* PCP, laggard	
***Relative advantage***	Mild positive opinion (early adopter, early majority)	Strong positive opinion (early adopter, early majority)
	*Eg. When it comes to community-based care for dependent elderly patients, it may help.* PCP, early adopter	*Eg. Patient monitoring is shared. This is a great help for community-based providers, especially because it’s a multidisciplinary team that can answer quite a lot of questions.* N, early majority
		*Eg. Until now, we’ve had a relationship with the attending physician of a subordinate to a higher hierarchical level. Now we have the impression that the relationship isn’t vertical, rather, it’s become more horizontal.* N, early adopter
		*Eg. As soon as there was a problem, we had to bother the general practitioner, who is already “overbooked.” But now I feel extremely safe, knowing that there are [geriatricians] above me, people whom I can ask for advice. I find this really incredible.* N, early majority
	Strong negative opinion (late majority, laggards).	Mild negative opinion (late majority, laggards)
	Eg. For me, [the CTM] has nothing to offer. We did just fine without it. PCP, laggard	*Eg. Who works 7 days a week? It’s the nurses, not the case managers. So I’m against imposing this person [case manager].* N, laggard

#### Different perceptions among PCPs and nurses

The differences in the diffusion curves between PCPs and nurses appear to be linked to different perceptions of the CTM’s *compatibility* and *relative advantage* (Tables 4 and 5).

Clinicians formed opinions on the *compatibility* of the CTM based on their own norms, values and practice and the roles that they wanted to play in the management of patient care. Even if, overall, the early adopters and early majority had positive views of the CTM’s *compatibility* with their own practices, the PCPs expressed more caution, especially regarding their role in the multidisciplinary team. In the late majority and laggard categories, PCPs had major concerns about their relationship with the case managers. They also had concerns about continuity of care, the sharing of information, their collaboration with specialists and the risk of decreased professional autonomy.

In terms of impact on patient management and practices, the nurses perceived a greater *relative advantage* to the CTM than the PCPs since they no longer found themselves dealing with complex situations alone. They were very pleased to see an opportunity for a non-hierarchical relationship with the PCPs, and were looking forward to collaborating closely with the PCPs. They also felt that communication with the geriatricians could facilitate their relationship with the PCP, especially when there was a disagreement on how to manage a patient.

### Dynamics of the diffusion process

The data gathered in the interviews allowed us to better understand how the CTM diffused among nurses and PCPs. It also revealed the importance of the concept of *critical mass* in this diffusion process. Indeed, the nurses very quickly attained a *critical mass* and the CTM was therefore systematically and quickly diffused. In contrast, much more time was required before a *critical mass* could be obtained among the PCPs (Figure 2). The interviews were particularly revealing, showing that the PCPs’ perceptions of the CTM’s features were not positive enough for them to immediately adopt it. Here, the roles of opinion leader or champion became critical (Table 6). The opinion leaders identified in this case were true innovators, and early in the implementation process, they were able to exercise influence over some PCPs and convince them to adopt the CTM. These PCPs became in turn early adopters. Throughout the implementation process, the innovators and the early adopters were able to convince the more resistant PCPs (late majority). The CTM was thus diffused in a slow snowball effect for PCPs.

**Table 6:** Dynamics of the diffusion process among Primary Care Physicians and Nurses

**Diffusion process**			**Evidence**
**Nurses (N)**	**Critical mass**	*Critical mass* attained very quickly	See Figure 2
	**Dynamics of the diffusion process**	Adoption of the CTM on the basis of its features alone	*I am thrilled with [the CTM] that they’re trying to show us. It’s innovative because it isn’t in the hospitals, where the hospital social workers are overwhelmed, that they’re doing it (…). It’s clear that [the CTM] really represents an attempt to do things the best way possible, to find solutions, treatments, tailored caregivers. This is where it’s really innovative. N, early majority*
**Primary Care Physicians (PCPs)**	**Critical mass**	More time was required before a *critical mass* could be obtained	See Figure 2
	**Dynamics of the diffusion process**	Early adopters (champion, opinion leaders) convince the early majority	*I believe that it was a long time ago that I was contacted by one of the [physicians]. And then it was off and running. PCP, early majority*
		Then, early adopters and early majority were able to convince the late majority	*I met with Dr. X, and I was impressed. I saw that [the CTM] could work very well. He made a very strong impression. PCP, late majority*
			*It’s good to have personal relationships with the doctors in charge of [the CTM], because personal contact is clearly a big help. It gives you an idea of who’s responsible, and if we get along, that makes it easier. So I tried it with one patient, just to see. PCP, late majority*
	**Role of the opinion leader**	Profile of the opinion leader: a PCP in the close social network; with similar practice and who has had a positive experience with the CTM	*In a way, we have the same background, and my colleague Dr. A is satisfied, too. He has many geriatric patients. Doctors with many geriatric patients need it more.* PCP, early majority
		Importance of the characteristics of the exchange between opinion leaders and other PCPs in terms of timeline and content	*I participated in [the model’s] development. I was contacted at the outset, before a team was put together. I know [physician] well.* PCP, early adopter
			*The administrative tasks related to management – those things that are not pure medicine – if I can be freed of them, I’d be thrilled.* PCP, early adopter

## Discussion

This study reported on the dynamic process by which a CTM was diffused among PCPs and nurses. The results highlighted the fact that adoption of a CTM by PCPs and nurses is a complex process and improved our understanding of the factors explaining adoption, or lack thereof, of a CTM. Indeed, we found that the PCPs adopted the CTM later and more slowly than the nurses. We identified the two key characteristics of the CTM explaining these differences in adoption dynamics between the two professional groups: (1) its relative advantage in terms of expected benefits for clinicians and patient care; and (2) its compatibility with the idiosyncrasies of primary care.

It is well known that CTMs are a potential response to the difficulties experienced by PCPs in caring for patients with multiple chronic diseases and may improve many care processes and health outcomes [9,10,27,28]. However, their adoption remains challenging. In principle, PCPs do favor greater participation of nurses in primary care delivery [29]. However, PCPs often resist CTM implementations as they have concerns about the threat to continuity of care and to their role as manager of their patients’ path through the health system [30,31]. Our results suggest that nurses adopt these models more quickly because they perceive a greater potential to improve quality of care and efficiency of practices and, at the same time, enhance their status and professional autonomy, which are key cultural values for nurses [32,33]. While there is evidence of a broad consensus about the importance of high-quality and efficient healthcare [34], the perceptions of how this can be achieved with the adoption of CTMs vary between PCPs and nurses.

This empirical study has advanced our understanding of the adoption and diffusion of CTMs in healthcare as a social phenomenon. Our results suggest that implementation of a CTM requires obtaining the collaboration of opinion leaders in order to obtain the commitment of PCPs. Given their critical role in the implementation of complex interventions, there is a growing interest in opinion leaders in the literature [35-40]. However, how to identify opinion leaders remains a challenge [40], and the process by which opinion leaders exert a positive influence has not yet been identified [41]. The results of this study provide some insight into the identity and role of opinion leaders. As in other study results [42], our results show that opinion leaders need to belong to the same professional group. In addition, we found that opinion leaders who had a similar type of practice and who had tested the CTM were particularly influential. Overall, our results suggest that opinion leaders are key at the beginning of a CTM’s implementation among PCPs. They speed up adoption by their colleague PCPs; in turn, these early adopters influence the adoption of the intervention by other PCPs, who represent the early and late majority. Through this dynamic process, a *critical mass* is reached and the intervention attains the point where diffusion becomes self-sustaining.

This study’s focus on one single region limits its generalizability. However, when considering different settings, the factors influencing diffusion may be more likely to vary in degree than in nature. In order to obtain a more complete view of the adoption of CTMs, it would be useful for future studies to include all professionals, e.g. specialists and social workers, and to have parallel studies conducted with patients. However, given the essential role of the nurse-PCP team, our results still provide critical information regarding the adoption of CTMs.

Notwithstanding its limitations, the strength of this study lies in the fact that it: (1) is longitudinal; (2) has a strong theoretical foundation and describes precisely how it was used [43]; (3) was conducted in a large geographical region; (4) uses two complementary sets of data: first the diffusion curves, which show different rates of adoption for PCPs and nurses, and second the qualitative study data, which deepen our understanding of the overall implementation process; and (5) includes diffusion data that clearly identify the actual date of adoption of the innovation – a rare occurrence [21].

This study also has practical implications. In order to successfully implement a new model of care, clinicians must be committed and be willing to take risks [44]. Fostering such commitment among clinicians requires complex and targeted strategies. In this perspective, inter-professional education is essential [45]. Moreover, there is a need to develop targeted strategies for CTM implementation. Early on, implementers need to identify opinion leaders among PCPs, people who have a positive attitude toward the CTM and will play an active leadership role in its planning and operationalization [27]. In addition, as clinicians act on the basis of “one trial = a definitive opinion about the CTM,” providing a “trialability space” and making the benefits of the CTM more visible may foster adoption [40]. Finally, since interventions fail when an insufficient number of clinicians adopt it [46], the concept of a *critical mass* of adopters may prove useful when developing and monitoring implementation strategies designed to accelerate the adoption process and maximize the benefits of CTMs.

## Conclusion

There are challenges in the actual implementation of CTMs, especially in terms of ensuring adoption by health professionals and, in particular, PCPs. Our results suggest that the diffusion of CTMs is influenced by two perceived key characteristics: (1) the CTM’s relative advantage in terms of expected benefits for clinicians and patient care; and (2) the CTM’s compatibility with the idiosyncrasies of primary care.

CTM diffusion is thus a social phenomenon, which requires a major commitment by clinicians; the role of opinion leaders is paramount. Paying attention to the notion of a *critical mass* of adopters is essential to developing implementation strategies that will accelerate the process by which health professionals adopt CTMs.

## Abbreviations

CTM: Collaborative team model; CTMs: Collaborative team models; PCPs: Primary care physicians; Nurse-PCP team: Nurse-Primary care physician team.

## Competing interests

All authors declare that they have no competing interest.

## Authors’ contributions

IV: concept and design, data acquisition and analysis, drafting of the manuscript, and revision of the manuscript. VG: data acquisition and analysis, and revision of the manuscript. MDS: data acquisition, study coordination and revision of the manuscript. CR: data acquisition and analysis, and revision of the manuscript. HB: concept and design, and revision of the manuscript. JA: concept and design, and revision of the manuscript. LL: concept and design, data acquisition and analysis, drafting of the manuscript, and revision of the manuscript. All authors read and approved the final manuscript.

## Funding statement

This study received financial support from the Canadian Institutes of Health Research (CIHR), the Haute Autorité en Santé - Caisse Nationale de Soldarité Autonomie (France) and the Dr. Joseph Kaufmann, Chair in Geriatric Medicine, McGill University. The sponsors played no role in the study design; the collection, analysis and interpretation of data; the writing of the manuscript; and the decision to submit the manuscript for publication.

## Pre-publication history

The pre-publication history for this paper can be accessed here:

http://www.biomedcentral.com/1471-2296/14/3/prepub

## Supplementary Material

Additional file 1 **Appendix. **Interview Guide. Click here for file
