# Supplementary material for: Diffusion of a collaborative care model in primary care: a longitudinal qualitative study
Source: BMC Fam Pract. 2013 Jan 4;14:3. doi: 10.1186/1471-2296-14-3 (PMC3558442; doi:10.1186/1471-2296-14-3)
Supplement: Additional file 1 — Appendix. Interview Guide. [file 1471-2296-14-3-S1.pdf]

Appendix: Interview Guide

When you did first learn about the collaborative team model (CTM)?
Examples of prompts and probing questions

How long you have known about the existence of the CTM
What were your first perceptions of the CTM?

- In terms of its simplicity or lack thereof (*Simplicity*)?
- In terms of its compatibility or lack thereof with how you see things, your experience, your expectations (*Compatibility*)?
- In terms of compatibility or lack thereof with your working practices (*Compatibility*)?
- Has it brought something new compared to the way things worked before (*Relative advantage*)?

Have you tried to use the CTM (*Triability*)?
Examples of prompts and probing questions

When?
Why?
Is there any factor that has influenced your participation?
What were your first perceptions when using the CTM?

Do you know any other health professionals who have tried to use it or who are using it (*Observability*)?
Examples of prompts and probing questions

What did you think about this?
What did they say about it?
Did they have any influence on your adoption of the CTM?

What do you see as the advantages and disadvantages of the CTM for healthcare professionals?
Examples of prompts and probing questions

Impact on your working practices
Impact on information exchange between healthcare professionals
Impact on care coordination
Impact on the decision-making process
Impact on task sharing between healthcare professionals

What do you see as the advantages and disadvantages of the CTM for the patient?

Impact on patient role?
Impact on quality of care?
